# Multi-semantic feature fusion attention network for binary code similarity detection

**DOI:** 10.1038/s41598-023-31280-w

**Published:** 2023-03-12

**Authors:** Bangling Li, Yuting Zhang, Huaxi Peng, Qiguang Fan, Shen He, Yan Zhang, Songquan Shi, Yang Zhang, Ailiang Ma

**Affiliations:** 1 Department of Security Technology, China Mobile Research Institute, Beijing, 100053 China; 2 Data & AI Technology Company, China Telecom Corporation Ltd, Beijing, 100011 China

**Keywords:** Computer science, Information technology

## Abstract

Binary code similarity detection (BCSD) plays a big role in the process of binary application security test. It can be applied in several fields, such as software plagiarism detection, malware analysis, vulnerability detection. Most research is based on recurrent neural networks, which is difficult to get the overall or long-distance semantic information of functions. Besides, exiting works simply extract high-level semantic features, lacking in-depth investigations on the potential mechanisms for fusing low-level and high-level semantic features. In this paper we propose a multi-semantic feature fusion attention network (MFFA-Net) for BCSD. MFFA-Net contains two critical modules: semantic feature fusion (SFF) and attention feature fusion (AFF). The SFF module concatenates multiple semantic features to represent the semantics of the function, which helps to obtain the overall semantic information of the function. The AFF module is designed to find useful information from various features, which assigns an attention matrix to research the relationship between features. In order to evaluate the proposed method, we made extensive experiments on two datasets. MFFA-Net can achieve a high degree of AUC at 99.6% and 98.3% respectively on the two datasets. The experimental results show that MFFA-Net has better performance for BCSD.

## Introduction

With the rise of the mobile internet, Internet of things (IoT) and 5G, complex software find applications in all kinds of new devices: the number of architectures running the same program has multiplied and COTS software components are increasingly integrated into closed-source products. This brings more security risks and challenges to terminals. For example, the extensive use of open source software for resource sharing leads to increased security risks; a large number of terminals are in an insecure physical environment, which is more likely to cause leakage of user and business sensitive information. SUMAP pointed out in the 2021 CVE Vulnerability Trend Security Analysis Report that the number of CVE statistics in 2020 has ranked first, and the number of Q1 CVEs in 2021 has reached an astonishing 13,000. Therefore, the automatic analysis of software artifacts in the compiled form (binary code) is of great significance. At the same time, finding similar functions in the compiled code segment gets the most attention.

**Figure 1:**
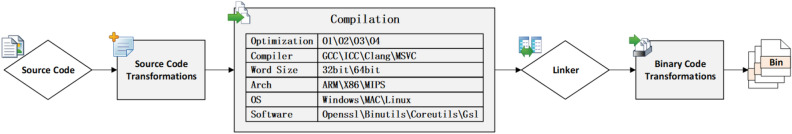
The extended compilation process.

The BCSD technique is used to measure the similarity relationship between two or more binary program components^1^. Depending on the detection granularity, program components can be basic blocks granularity, functions granularity, or entire programs. Binary code similarity can be applied to scenarios such as software plagiarism detection^2,3^, malware detection and analysis^4–6^, vulnerability detection^7^. One of the main challenges of BCSD is that the same source code will be compiled into different binary codes after using different versions of compilers and selecting different compilation options, etc. As shown in Fig. 1, modifying any gray box in the figure will make the same source code compiled into different but semantically equivalent binary programs. This will happen all the time. For example, in order to improve the efficiency of the program, the version of the compiler may be changed, or the compiler may be changed completely. In order to be suitable for different architectures, the target platform can be changed. What’s more, it is possible to deliberately apply obfuscating transformations to generate polymorphic variants of the same source code. An ideal goal of BCSD is that they can identify the similarity of binary codes corresponding to the same source code that has undergone different conversions.

In this paper, we propose a novel architecture MFFA-Net, to detect binary code similarity. We propose a SFF module, introducing shallow features and MLP networks to learn global structural features. We put forward the AFF module, studying the feature fusion method in depth. The relationship between the features is captured through the attention matrix, and the attention mechanism is added to the features for feature fusion to extract more refined features. MFFA-Net aims to extract overall structural features and finer features without reducing efficiency.

## Related works

### Traditional binary code similarity detection

Considering its applications and challenges, many binary code similar methods have been proposed. Traditional methods mainly include Binhunt^8^, iBinHunt^9^, BinClone^10^, Multi_MH^11^, discovRE^12^. etc. BinHunt employed a new graph isomorphism technique, symbolic execution, and theorem proving to identify the semantic differences. iBinHunt utilized deep taint to identify semantic differences in control flow between programs, but still had low accuracy and high overheads. BinClone used hashing to obtain a fixed-length value out of a variable-length instruction sequence and represents a piece of binary code as a bit-vector to compute similarity. Multi_MH was the first cross-architecture binary code search method. It indexed functions based on input and output semantics. For example, given a function compiled for a CPU architecture (such as x86), Multi_MH can find similar functions compiled for other architectures. DiscovRE used the number of arithmetic instructions and call instructions of the basic block as features, and uses a backtracking algorithm to repair incorrect matches. However, these algorithms are time-consuming and difficult to deal with a large number of function pairs. David et al. developed a tool called Esh^13^ and its successor GitZ^14^, which used for large-scale detection with high accuracy. Chandramohan el al.^15^ proposed a scalable and robust binary search engine called Bingo that captured the full function semantics by inlining related libraries and user-defined functions. They have the disadvantage that the false positive rate is high in the cross-optimization option scenario and dynamic analysis is required to cooperate with static analysis.

### Deep learning-based binary code similarity detection

Methods based on deep learning are divided into end-to-end detection methods and multi-stage detection methods. The end-to-end detection methods mainly include αDiff^16^, Asm2vec^17^, CodeCMR^18^, etc. In order to avoid manually selected features, these methods directly extract features using instructions or raw bytes. The multi-stage detection methods include feature selection and feature encoding. Feng et al.^19^ proposed a bug search approach Genius, which first used embedding vectors for feature selection. Xu et al.^20^ proposed a novel neural network-based approach Gemini for similarity detection. Gemini uses a graph embedding model to embed the control flow graph (CFG) of a function. Zhu et al.^21^ proposed a similar detection method SimInspector based on neural machine translation (NMT) and graph embedding. They validated that SimInspector is about 6% higher than that of Gemini in similarity detection accuracy rate. Zuo et al.^22^ implemented a prototype system INNEREYE for large-scale binary code analysis. They used embeddings for natural language processing (NLP) to encode categorical features. BinDeep, a hybrid model, was proposed by Tian et al.^23^. BinDeep used RNN to identify the specific types of two functions and used the siamese neural networks to calculate the similarities of two functions. Although embedding can automatically learn features, the embedding function does not provide any information about the learning content. Massarelli et al.^24^ proposed a novel architecture SAFE that directly extracted function features based on assembly instructions and achieves high performance. They added some application scenarios based on this article and published it in 2021^25^. SAFE architecture does not incur in the computational overhead of building or manipulating control flow graphs, and this leads to a considerable speed advantage, and it's more general as it works on stripped binaries and on multiple architectures. But there are still some needs to be be improved. In their model, the latter words have a greater impact on the results than the previous words. When it is used to capture the semantics of the entire function, the efficiency will be reduced and the effect will be worse.

## Problem definition and solution overview

### Problem definition

If the same source code *s* is changed in the gray part as shown in Fig. 1, similar binary files will be disassembled to obtain similar binary functions. This paper only considers similar functions under different compilers. The similar binary functions $f_{1} ,f_{2} , \ldots ,f_{n}$ are the result of compiling the same original source code $s$ with different compilers. The compiler $c$ maps a source code $s$ to the corresponding binary function $f$. A specific software is considered as a compiler in this paper.

We use $I_{{f_{1} }} :(l_{1} ,l_{2} ,l_{3} , \ldots ,l_{m} )$ to represent the assembly instructions list that composes the function $f_{1}$ where $m$ represents the number of instructions. During data processing, $f_{1}$ is represented as a vector in ${\mathbb{R}}^{n}$. An embedding vector $\overrightarrow {{f_{1} }} \in {\mathbb{R}}^{n}$, which is obtained by mapping $I_{{f_{1} }}$, retains the similarity structure of the binary functions.

### MFFA-Net overview

**Figure 2:**
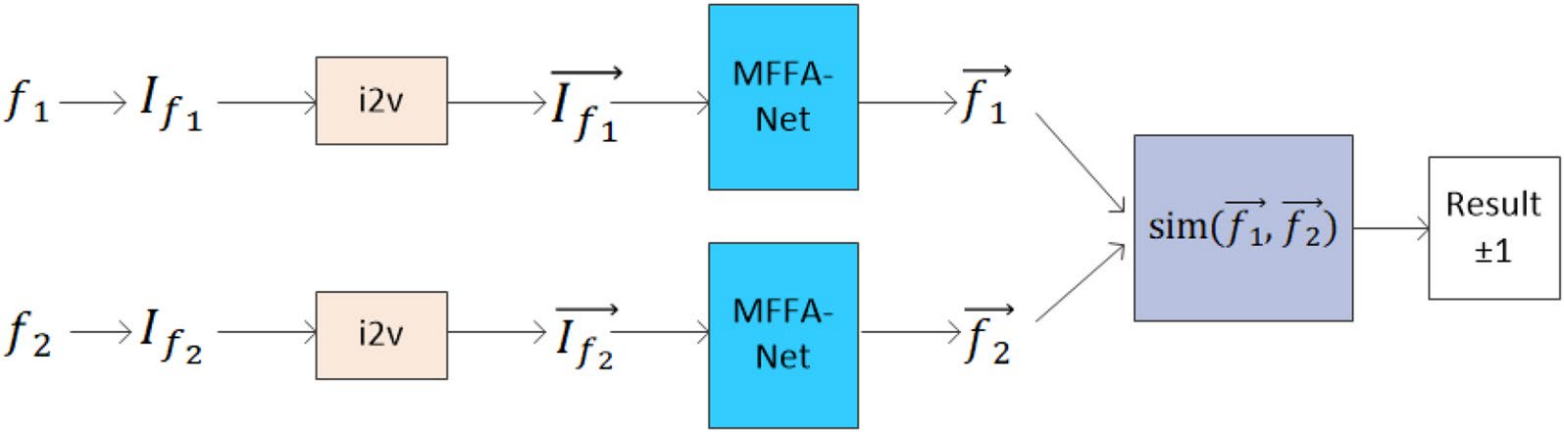
The process of identifying the similarity between two binary functions.

The process of identifying the similarity between two binary functions is shown in Fig. 2. The MFFA-Net-based embedding model takes a pair of binary functions as inputs, and computes a similarity score as the output. There are two steps in which an embedded model structure is used. In the first step, the *Assembly Instructions Embedding* component converts a sequence of assembly instructions $I_{{f_{1} }}$ into a sequence of vectors. In the second step, MFFA-Net converts a sequence of vectors in a single embedding vector.

- **Assembly instructions embedding** Each instruction $l \in I_{f}$ is mapped to a vector of real numbers $\vec{l}$, using the instruction2vec (i2v) model that trained through a large corpus of instructions. A sequence of vectors $\overrightarrow {{I_{f} }}$ is the final output in this step.
- **MFFA-Net** There are two modules in this network. The first is the semantic feature fusion (SFF) module, which concatenating input feature and the advanced semantic features to remedy the lack of overall semantic information caused by incomplete feature information captured by RNN. The advanced semantic features compute a summary vector taking into account the instruction itself and its context in $I_{f}$, using a bi-directional recurrent neural network. The other is the attention feature fusion (AFF) module, which is designed to find useful information from various features and research the relationship between features.

## Details of the MFFA-Net

**Figure 3:**
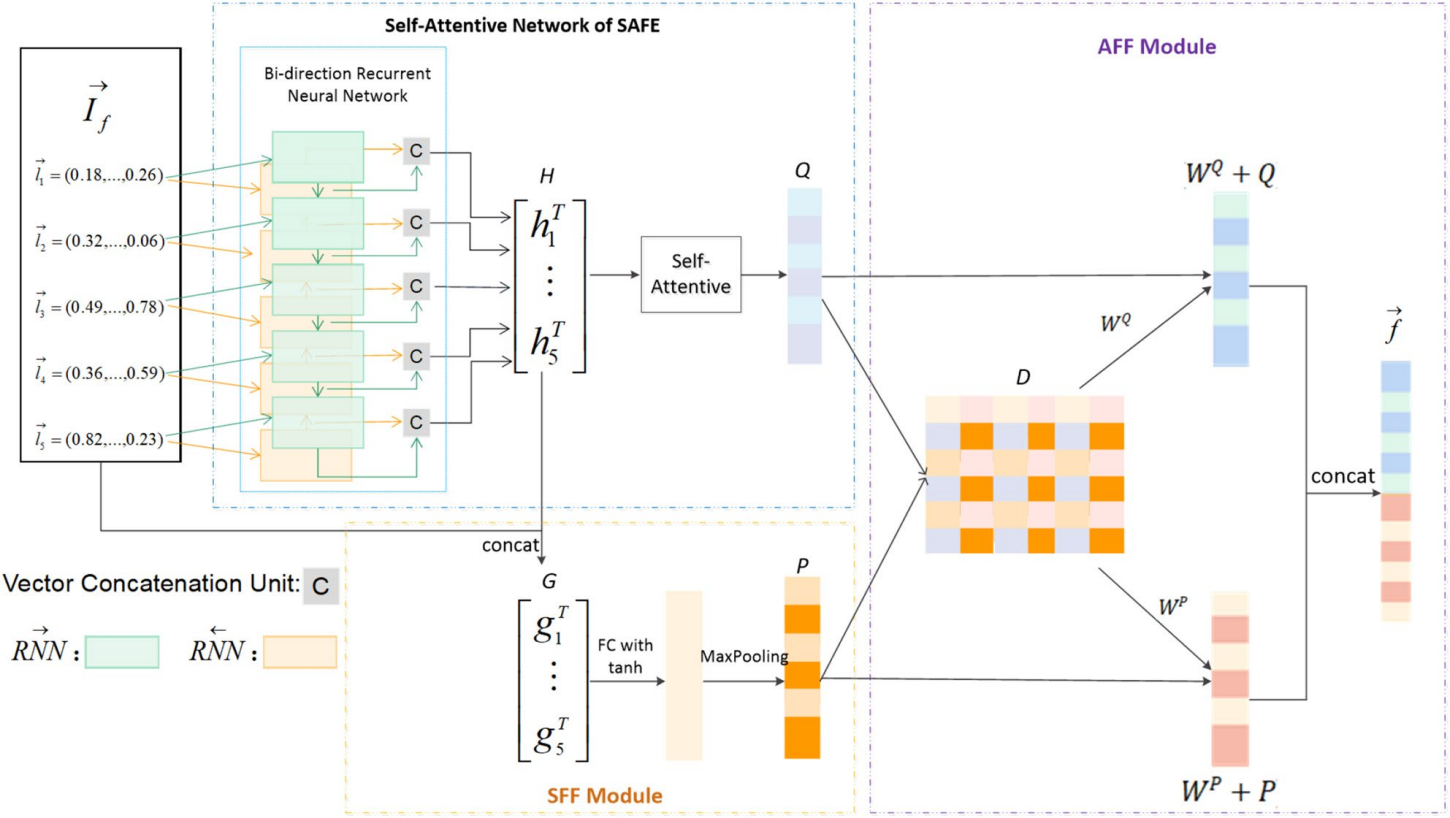
The overall framework of MFFA-Net.

Our model is based on SAFE^24^. The input of our model is the binary code functions’ instructions. The overall structure of our model is shown in Fig. 3. On the advanced semantic feature extraction module, the model takes the word embedding as input and uses SAFE to extract function embedding. On the SSF module, the model concatenates the advanced semantic feature and word embedding feature to learn multi scale feature, and then uses fully connect (FC) layer and $Maxpooling$ layer to extract SSF feature. On the AFF module, matrix $D$ is computed by advanced semantic feature and the SSF feature, which presents the interaction between features. The model uses interaction attention to compute weight of features, and finally concatenates the weighted feature to compute the function embedding.

### Semantic feature fusion module

In SFF module, we concatenate word embedding, which helps us to obtain more overall information of a function, and high-level feature, which is extracted by bi-directional recurrent neural network and helps us to capture the contexts of a function.

As shown in Fig. 3, the sequence of instruction vectors $\overrightarrow {{I_{f} }} :(\overrightarrow {{l_{1} }} ,\overrightarrow {{l_{2} }} ,\overrightarrow {{l_{3} }} , \ldots ,\overrightarrow {{l_{m} }} )$, which is achieved by training i2v model through a large corpus of instructions, is fed into a bi-directional neural network. For each $\overrightarrow {{l_{1} }} \in \overrightarrow {{I_{f} }}$ obtaining a summary vectors of size $u$:

$$h_{i} = \overrightarrow {RNN} (\overrightarrow {{h_{i - 1} }} ,\overrightarrow {{l_{i} }} ) \oplus \overleftarrow {RNN} (\overleftarrow {{h_{i + 1} }} ,\overrightarrow {{l_{i} }} )$$

where $\oplus$ is the concatenation operand, $\overrightarrow {RNN}$ and $\overleftarrow {RNN}$ represent the forward and backward RNN cell, respectively. $\overrightarrow {{h_{i - 1} }}$ and $\overleftarrow {{h_{i + 1} }}$ are the forward and backward states of the RNN $(\overrightarrow {{h_{i - 1} }} = \overleftarrow {{h_{i + 1} }} = 0)$. The state of each RNN cell has size $u/2$. The instruction embedding $\overrightarrow {{l_{i} }}$ has size $d$. Then concatenating the input instruction vectors and $h_{i}$, we can get a summary vector of size $u + d$:

$$g_{i} = h_{i} \oplus \overrightarrow {{l_{i} }}$$

We obtain a $m \times (u + d)$ matrix $G$ from above summary vectors. Then through an MLP network we constructed, the matrix G is transformed into a fusion feature embedding $P$. Using a FC layer with $tanh$ activation and a global $Maxpooling$ layer to compute $P$ of size $n$:

$$P = Maxpooling(tanh(G))$$

The fully connected layer can learn the overall semantic information of the function, and then perform the $Maxpooling$ operation to reduce information loss and retain more characteristic information.

### Attention feature fusion module

In AFF module, for in-depth study of the relationship between features, we extract a cross matrix $D$. Each point in the cross matrix $D$ records the interaction factor between features.

Firstly, $\overrightarrow {{I_{f} }}$ is used to compute the feature embedding $Q$ of size $n$ through the Self-Attentive Network of SAFE method. The cross matrix $D$ of size $n \times n$ is computed by $P$ from the SFF module and $Q$:

$$D = QP^{T}$$

The attention weight $W^{P} ,W^{Q}$ are compute as follow:

$$\begin{aligned} W^{P} & = softmax(P \times (w^{P} \times ||D||_{2} )) \\ W^{Q} & = softmax(Q \times (w^{Q} \times ||D||_{2} )) \\ \end{aligned}$$

where $w^{P}$, $w^{Q}$ are the weight matrix of size $n \times n$. $w^{P}$ is a weight acted on $P$, $w^{Q}$ is a weight acted on $Q$. $||C||_{2}$ is $L_{2}$ Normalization for the cross matrix $D$. Finally, fusion the features with attention weight to get the embedding vector $\vec{f}$ of size $2 \times n$:

$$\vec{f} = (W^{P} + P) \oplus (W^{Q} + Q)$$

where $\oplus$ is the concatenation operand. The attention weight $W^{P} ,W^{Q}$ represent the degree of weakening or increasing the feature. The input feature vector is added to the attention weight to obtain the weighted feature vector, which makes the feature vector expression more accurate.

Our goal is to make sure the same source code has higher similarity scores than others. Same as SAFE, use a *siamese*^26^ network on our embedding model to reduce loss and use cosine distance to compute the similarity. For two binary functions $f_{1}$, $f_{2}$, $\overrightarrow {{f_{1} }}$, $\overrightarrow {{f_{2} }}$ are obtained by using the i2v and MFFA-Net model. The cosine similarity of $\overrightarrow {{f_{1} }}$, $\overrightarrow {{f_{2} }}$ is calculated by following formula:

1 $$sim(\overrightarrow {{f_{1} }} ,\overrightarrow {{f_{2} }} ) = \frac{{\mathop \sum \nolimits_{i = 1}^{n} (\overrightarrow {{f_{1} }} [i] \cdot \overrightarrow {{f_{2} }} [i])}}{{\sqrt {\mathop \sum \nolimits_{i = 1}^{n} (\overrightarrow {{f_{1} }} [i])} \cdot \sqrt {\mathop \sum \nolimits_{i = 1}^{n} (\overrightarrow {{f_{2} }} [i])} }}$$

where $\vec{f}[i]$ is the *i*-th component of the $\vec{f}$. $f_{1}$, $f_{2}$ are similar if $sim(\overrightarrow {{f_{1} }} ,\overrightarrow {{f_{2} }} ) \to 1$; otherwise, $f_{1}$, $f_{2}$ are dissimilar if $sim(\overrightarrow {{f_{1} }} ,\overrightarrow {{f_{2} }} ) \to - 1$.

## Experiment

All experiments are implemented in TensorFlow in Python version 3.6.9. The optimizer is Adam. Dimension of the function embedding size is 200. We train our models for 30 epochs. The optimal setting for our model: learning rate is 0.001, batch size is 250. The other parameters are the same as set in SAFE. Detailed configuration information is as follows:

Computer configuration: Ubuntu 18.04 (64-bit), 16 GB of memory, Intel(R) Core(TM) i7-6700T CPU@2.80GHz; GPU: GeForce GTX 950A, 2 GB; TensorFlow version: 1.5.0.

### Datasets

**Figure 4:**
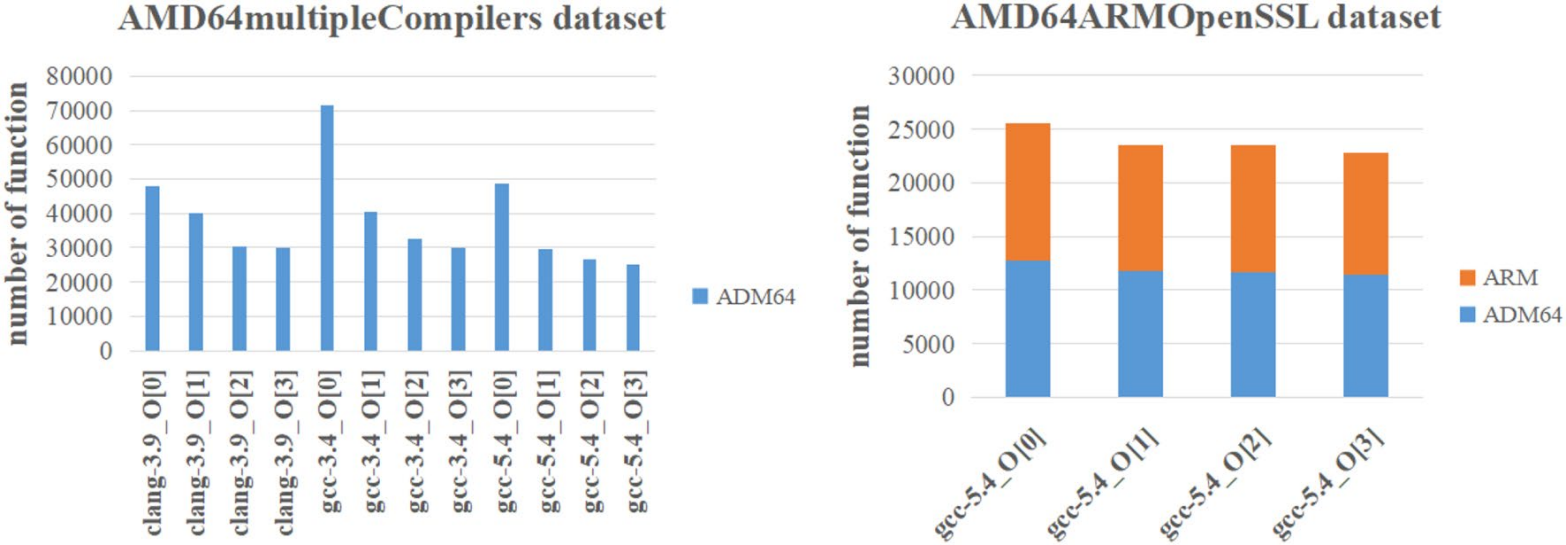
Datasets analysis.

Two datasets^24^ are used to verify the effectiveness of our method. One is AMD64ARMOpenSSL dataset, which has 95,535 functions compiled for the cross-platform AMD64 and ARM. The other is AMD64multipleCompilers dataset, which has 452,598 functions compiled for the single platform AMD64. The AMD64ARMOpenSSL dataset is generated by using one compiler (gcc-5.4) with 4 optimization levels (-O[0-3]). The AMD64multipleCompilers dataset is generated by using three compilers (clang-3.9, gcc-5.4, gcc-3.4) with 4 optimization levels (-O[0-3]). The basic statistics of datasets are shown in Fig. 4.

Two pairs, which are a similar pair and a dissimilar pair, are created for each function in two datasets. Then the total number of pairs finally obtained is twice the total number of functions. The similar pairs are labeled as + 1. The dissimilar pair are labeled as − 1.

We generate our training and test pairs as described in Literature 24. The functions in AMD64multipleCompilers dataset are divided into 14:3:3 as training, validating and testing set, i.e., 70% for training, 15% for validating and 15% for testing. The functions in AMD64ARMOpenSSL dataset are divided into 4:1 as training and testing set, i.e., 80% for training and 20% for testing.

### Evaluation metrics

The Receiver Operating Characteristic (ROC) curve was chosen as the key performance indexes^24^. The area under curve (AUC), where the higher AUC the better, is the area under the ROC curve^27^. We also chose another important precision-recall (PR) curve as an evaluation index. The PR curve, which was first used by Raghavan et al.^28^ in information retrieval, has become more popular in recent years. The area under PR curve is called the average precision (AP)^29^. When comparing the prediction performance of the two new and old models, if the ROC curve and PR curve of the new model are better than the old model at the same time, then the performance of the new model is considered to be better than the old model^30^.

### Experimental results

We compared our method with the SAFE method and used two evaluation metrics, AUC and AP, for verification. First of all, we reproduce the effect of the SAFE method on the two datasets. The effect on the AMD64ARMOpenSSL dataset is lower than that in Literature 24 because we did not use cross-validation training for quantitative comparison with the multi result. At the same time, time consumption of the model is also considered. The experimental results are shown in Table 1.

- **The results on AMD64multipleCompilers dataset** In the single-platform case, our model MFFA-Net get high scores in two metrics (99.6% in AUC, 99.6% in AP) from Table 1. The AUC and AP values of the SAFE model are 98.8% and 98.8%, respectively. The MFFA-Net model is 0.8% higher in terms of AUC and AP than the SAFE model. The training time of the MFFA-Net model is 660 min, which is much less than the 1050 min of the SAFE model. There are 127,076 pairs of functions in the test set that need to be embedding. The inference time of the MFFA-Net model is 23 s longer than the 88 s of the SAFE model.
- **The results on AMD64ARMOpenSSL dataset** In the cross-platform case, in terms of AUC and AP, which are considered the most crucial metrics for binary classification, MFFA-Net outperforms SAFE by one percentage point. The AUC and AP values of the SAFE model are 97.1% and 97.3%, respectively. The AUC and AP values of our model are 98.3% and 98.1%, respectively. The training time of the MFFA-Net model is 180 min, which is much less than the 290 min of the SAFE model. The time taken by the MFFA-Net model to infer 19,140 pairs of functions in the test set is 14 s, which is 1 s longer than the time taken by the SAFE model.

**Table 1:** Comparison results using different models on two datasets.

Model	AMD64multipleCompilers dataset				AMD64ARMOpenSSL dataset			
	AUC	AP	training time (min)	inference time (s)	AUC	AP	training time (min)	inference time (s)
SAFE	0.988	0.988	1050	**88**	0.971	0.973	290	**13**
MFFA-Net	**0.996**	**0.996**	**660**	111	**0.983**	**0.981**	**180**	14

The inference time is the time consumed in the whole process from the input of the instruction vector sequence to the output of the similarity result in Fig. 2. *min* minute, *s* second.

Significant values are in bold.

**Figure 5:**
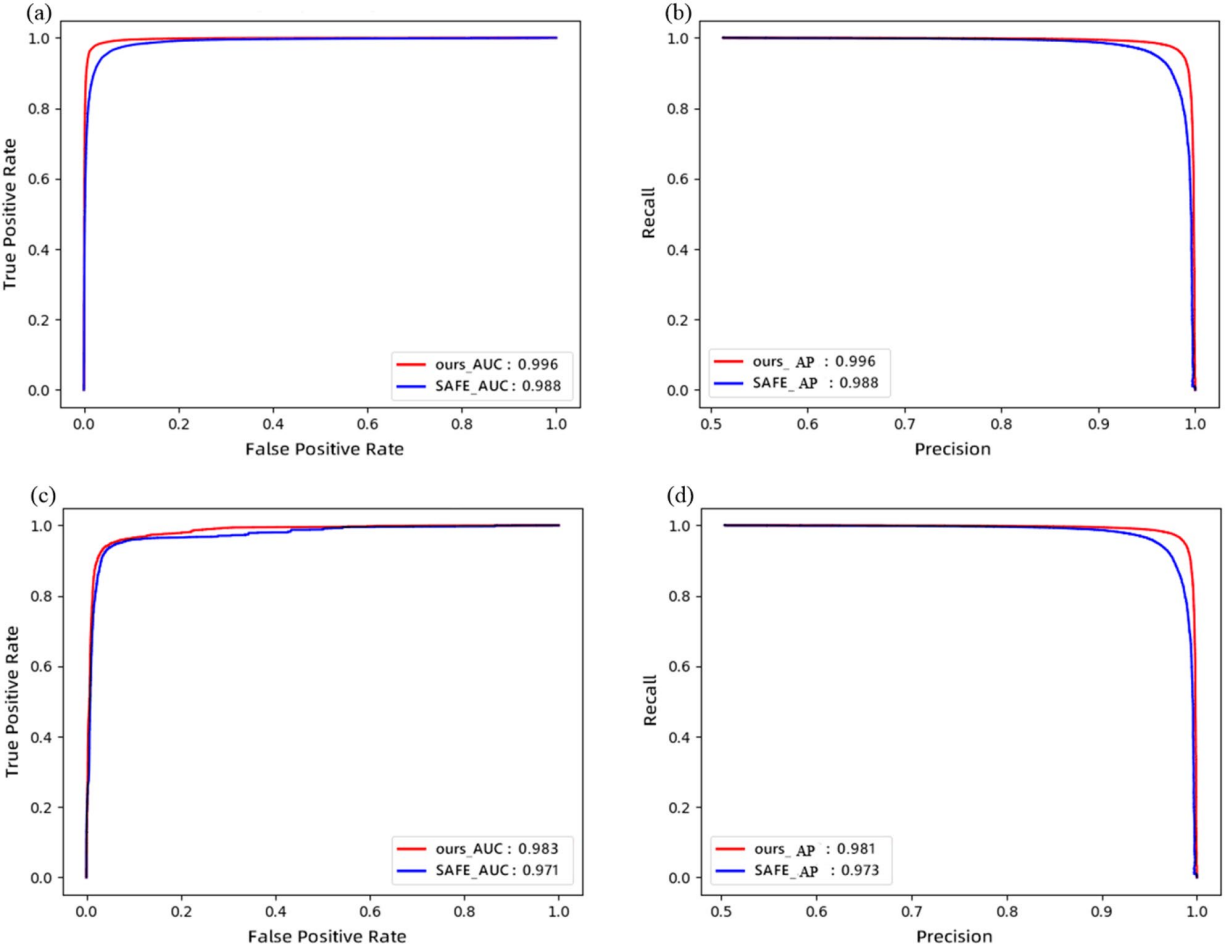
Performance comparison on the test set. (**a**) ROC curves on AMD64multipleCompilers Dataset; (**b**) PR curves on AMD64multipleCompilers Dataset; (**c**) ROC curves on AMD64ARMOpenSSL Dataset; (**d**) PR curves on AMD64ARMOpenSSL Dataset.

**Figure 6:**
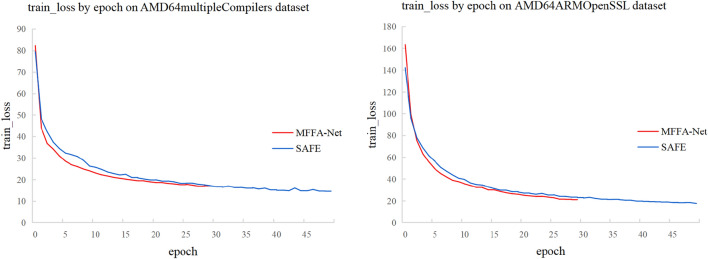
Training loss comparison.

In Fig. 5, we can see that the ROC and PR curves of the MFFA-NET model completely cover those of the SAFE model, indicating that MFFA-NET performs better than SAFE. Figure 6 shows the comparison results of training losses between the two models. As can be seen from Fig. 6, the convergence speed of MMFA-Net is faster than that of SAFE, which also proves that the training speed of MFFA-Net is faster.

Regardless of whether it is a single-platform case or a cross-platform case, the AUC and AP values of the MFFA-Net model are better than those of the SAFE model. Compared with SAFE, the total consumption of training time and inference time of MFFA-Net model is much smaller. It seems that the ability for BCSD of our model is efficient.

## Conclusion

We propose a new approach MFFA-Net that has better capability for BCSD in the single-platform or cross-platform case. MFFA-Net can not only learn overall structural features, but also deeply dig into the relationships between features. To evaluate this method we proposed, two datasets are used in our experiments. Experimental results in Fig. 5 and in Table 1 demonstrate that the MFFA-Net model is valid for BCSD. The MFFA-Net method has a high score overall AUC and AP. Compared with SAFE, MFFA-Net can achieve better performance in different metrics. As can be seen from Table 1, MFFA-Net has a great advantage in training time. In future research, we will continue to optimize and strive to make the inference time shorter. We also will try to apply MFFA-Net to other application scenarios, such as advanced persistent threat (APT) classification, semantic classification, etc.

## Data Availability

The AMD64multipleCompilers dataset and the AMD64ARMOpenSSL dataset are described in Massarelli et al.^24^ and can be found at https://github.com/gadiluna/SAFE.

## References

[1] Haq, I. U. & Caballero, J. *A survey of binary code similarity*. https://arxiv.org/abs/1909.11424 (2019).

[2] Luo, L., Ming, J., Wu, D., Liu, P. & Zhu, S. Semantics-based obfuscation-resilient binary code similarity comparison with applications to software plagiarism detection. In *International Symposium on Foundations of Software Engineering* 389–400 (ACM, 2014).

[3] Luo L, Ming J, Wu D, Liu P, Zhu S (2017). Semantics-based obfuscation-resilient binary code similarity comparison with applications to software and algorithm plagiarism detection. IEEE Trans. Softw. Eng..

[4] Lindorfer, M., Federico, A. D., Maggi, F., Comparetti, P. M. & Zanero, S. Lines of malicious code: Insights into the malicious software industry. In *Annual Computer Security Applications Conference* 349–358 (ACM, 2012).

[5] Cesare S, Xiang Y, Zhou W (2014). Control flow-based malware variant detection. IEEE Trans. Dependable Secure Comput..

[6] Farhadi, M. R. *et al.* Scalable code clone search for malware analysis. In *Digital Investigation the International Journal of Digital Forensics & Incident Response 15 (DEC)* 46–60 (2015).

[7] Gao, J., Yang, X., Fu, Y., Jiang, Y. & Sun, J. VulSeeker: A semantic learning based vulnerability seeker for cross-platform binary. In *Automated Software Engineering* 896–899 (ACM, 2018).

[8] Gao, D., Michael, K., Reiter, M. K. & Song, D. 2008. Binhunt: Automatically finding semantic differences in binary programs. In *International Conference on Information and Communications Security* 238–255 (2008).

[9] Ming, J., Pan, M. & Gao, D. iBinHunt: Binary hunting with inter-procedural control flow. In *International Conference on Information Security and Cryptology* 92–109 (2012).

[10] Farhadi, M. R., Fung, B. C. M., Charland, P. & Debbabi, M. BinClone: Detecting code clones in malware. In *Software Security and Reliability* 78–87 (2014).

[11] Pewny, J., Garmany, B., Gawlik, R., Rossow, C. & Holz, T. Cross-architecture bug search in binary executables. In *2015 IEEE Symposium on Security and Privacy* 709–724 (2015).

[12] Eschweiler, S., Yakdan, K. & Gerhards-Padilla, E. discovRE: Efficient cross-architecture identification of bugs in binary code. In *Network and Distributed System Security Symposium*. 10.14722/ndss.2016.23185 (2016).

[13] David Y, Partush N, Yahav E (2016). Statistical similarity of binaries. ACM SIGPLAN Not..

[14] David Y, Partush N, Yahav E (2017). Similarity of binaries through re-optimization. ACM SIGPLAN Not..

[15] Chandramohan, M. *et al.* Bingo: Cross-architecture cross-os binary search. In *Proceedings of the 2016 24th ACM SIGSOFT International Symposium on Foundations of SoftwareEngineering* 678–689 (ACM, 2016).

[16] Liu, B. *et al.* αDiff: Cross-version binary code similarity detection with DNN. In *Conference on Automated Software Engineering* 667–668 (ACM, 2018).

[17] Ding, S., Fung, B. & Charland, P. Asm2vec: Boosting static representation robustness for binary clone search against code obfuscation and compiler optimization. In *IEEE Symposium on Security and Privacy* (IEEE Computer Society, 2019).

[18] Yu, Z. *et al.* Order matters: Semantic-aware neural networks for binary code similarity detection. In *Proceedings of the AAAI Conference on Artificial Intelligence*, Vol. 34 1145–1152. 10.1609/aaai.v34i01.5466 (2020).

[19] Feng, Q. *et al.* Scalable graph-based bug search for firmware images. In *Computer and Communications Security* 480–491 (ACM, 2016). 10.1145/2976749.2978370.

[20] Xu, X. *et al.* Neural network-based graph embedding for cross-platform binary code similarity detection. In *Computer and Communications Security* 363–376 (ACM, 2017). 10.1145/3133956.3134018.

[21] Zhu X, Jiang L, Chen Z (2021). Cross-platform binary code similarity detection based on NMT and graph embedding. Math. Biosci. Eng..

[22] Zuo, F. *et al.* Neural machine translation inspired binary code similarity comparison beyond function Pairs. In *Network and Distributed System Security Symposium*. 10.14722/ndss.2019.23492 (2019).

[23] Tian D, Jia X, Ma R, Liu S, Liu W, Hu C (2021). BinDeep: A deep learning approach to binary code similarity detection. Expert Syst. Appl..

[24] Massarelli, L., Luna, G. A. D., Petroni, F., Baldoni, R. & Querzoni, L. SAFE: Self-attentive function embeddings for binary similarity. In *Detection of Intrusions and Malware, and Vulnerability Assessment* 309–329 (Springer, 2019).

[25] Massarelli L, Luna GAD, Petroni F, Querzoni L, Baldoni R (2021). Function representations for binary similarity. IEEE Trans. Dependable Secure Comput..

[26] Bromley, J., Guyon, I., LeCun, Y., Säckinger, E. & Shah, R. Signature verification using a “siamese” time delay neural network. In *Neural Information Processing Systems* 737–744 (ACM, 1993).

[27] Fawcett T (2006). An introduction to ROC analysis. Pattern Recognit. Lett..

[28] Raghavan V, Bollmann P, Jung GS (1989). A critical investigation of recall and precision as measures of retrieval system performance. ACM Trans. Inf. Syst..

[29] Zhou QM, Zhe L, Brooke RJ, Hudson MM, Yuan Y (2021). A relationship between the incremental values of area under the ROC curve and of area under the precision-recall curve. Diagn. Progn. Res..

[30] Davis, J. & Goadrich, M. The relationship between precision-recall and roc curves. In* Proceedings of the 23rd International Conference on Machine Learning* 233–240 (ACM, 2006).

